# Two new hybrids of the genus *Diplazium* (Athyriaceae) from Japan

**DOI:** 10.3897/phytokeys.172.60660

**Published:** 2021-02-01

**Authors:** Kiyotaka Hori, Hironobu Kanemitsu

**Affiliations:** 1 The Kochi Prefectural Makino Botanical Garden 4200-6 Godaisan, Kochi 781-8125, Japan The Kochi Prefectural Makino Botanical Garden Kochi Japan; 2 IDEA Consultants, Inc., 1-5-12 Higashihama, Higashi-ku, Fukuoka City, Fukuoka 812-0055, Japan IDEA Consultants, Inc. Fukuoka Japan

**Keywords:** Athyriaceae, *
Diplazium
*, Japan, new hybrid

## Abstract

In this study, we describe the ferns Diplazium × kanayamaense hyb. nov. and D. × tsukushiense hyb. nov. and further compare them to parental species *D. chinense*, *D. deciduum* and *D. fauriei* in terms of morphological characteristics, plastids and nuclear DNA markers. These new hybrids have been determined to be endemic to western Japan. The International Union for Conservation of Nature and Natural Resources status was evaluated for D. × kanayamaense as endangered (EN) and D. × tsukushiense as critically endangered (CR).

## Introduction

One of the important characters of Japanese fern flora is the richness of hybrids. Japanese pteridologists have been recognising morphological variations of Japanese ferns and they recognised many hybrids confuse identification of Japanese ferns (Tagawa 1959; Nakaike 1992; Ebihara 2017). However, some Japanese hybrid ferns still are not described in Latin or English languages and they do not have scientific names. Especially, Athyriaceae has still many undescribed hybrids (Ebihara 2017) and there is still not enough evidence to support a combination of their hypothesised parents.

*Diplazium* has been identified as the largest genus of Athyriaceae (PPG I 2016). It reportedly has 300–400 species (Wei et al. 2013; Liu et al. 2018), of which 26 species, eight varieties and 25 hybrids are recorded in Japan. Of these, 15 hybrids still do not have scientific names (Ebihara 2017). Hori and Murakami (2019) reported reticulate evolution of apogamous and sexual species in the *D. hachijoense* complex and found four undescribed apogamous species, based on plastid and nuclear markers. However, describing these ferns has been difficult as DNA phylogenies suggest that several undetected species are present in this complex. Otherwise, providing a description for these hybrids is easy when parents are endemic to Japan. In this study, we focused on D. × toriianum Sa.Kurata, *D. mettenianum* complex (Ohta and Takamiya 1999) and *D. chinense* (Baker) C.Chr.

Kurata (1960) has described D. × toriianum [*D. mettenianum* (Miq.) C.Chr.× *D. squamigerum* (Mett.) Matsum.] in terms of morphological characteristics. Subsequently, Ohta and Takamiya (1999) have recognised that the Japanese *D. mettenianum* complex contains *D. deciduum* N.Ohta & M.Takamiya sp. nov., *D. fauriei* Christ, *D. griffithii* T.Moore, *D. hayatamae* N.Ohta & M.Takamiya and *D. mettenianumsensu stricto*, based on cytology. Parents of D. × toriianum are likely to be *D. deciduum* and *D. mettenianum* (Ebihara 2017).

Meanwhile, Tsutsui (1988) found one undescribed fern from Kyushu, Japan. He considered the fern to be a hybrid of *D. chinense* and *D. fauriei*, based on its morphological characteristics; this claim was supported by Ebihara (2017). Further, Kanemitsu (2019) has also found one undescribed fern nearby that might be a hybrid of *D. chinense* and *D. deciduum*. However, both Tsutsui (1988) and Kanemitsu (2019) did not provide scientific names with appropriate descriptions. Thus, this study identifies two new hybrids, D. × kanayamaense (*D. chinense* × *D. deciduum*) and D. × tsukushiense (*D. chinense* × *D. fauriei*), with descriptions based on morphological characteristics, plastids and nuclear DNA markers.

## Materials and methods

### Plant materials and DNA extraction

Total DNA for molecular analyses was extracted from silica-dried leaves using cetyltrimethylammonium bromide, as previously described (Doyle and Doyle 1990).

*Diplazium chinense*, *D. squamigerum*, D. × kanayamaense, D. × tsukushiense, D. × toriianum, members of the *D. mettenianum* complex (*D. deciduum*, *D. fauriei*, *D. hayatamae*, *D. mettenianum* and *D. griffithii*) and several additional species of *Diplazium* (*D. amamianum*, *D. donianum*, *D. esculentum*, *D. nipponicum*, *D. takii* and *D. wichurae*) were examined using molecular DNA analysis. We used four species of the genus *Deparia* as an outgroup (*De. japonica*, *De. lancea*, *De. unifurcata* and *De. viridifrons*). Voucher information for all samples is provided in Appendix 1. All voucher specimens are deposited in the herbarium of the Kagoshima University Museum (**KAG**), Tokyo Metropolitan University (**MAK**), the Kochi Prefectural Makino Botanical Garden (**MBK**) or the National Museum of Nature and Science (**TNS**).

Additionally, we considered specimens from the Collection Database and Materials of TNS (http://db.kahaku.go.jp/webmuseum/), PE (http://pe.ibcas.ac.cn/en/), TAIF (http://taif.tfri.gov.tw/search.php), JSTOR Global Plants (https://plants.jstor.org/) and the Global Biodiversity Information Facility (https://www.gbif.org) database.

For conservation assessment, area of occupancy (AOO) and extent of occurrence (EOO) were estimated using GeoCAT (Bachman et al. 2011); default settings for grid size were also applied.

### Plastid and nuclear DNA sequencing

We sequenced plastid *trnL-F* and nuclear *AK1* gene following methods from Hori and Murakami (2019), but with modified conditions for polymerase chain reaction–single-strand conformation polymorphism (PCR-SSCP) analysis of the nuclear *AK1* gene. Electrophoresis of *AK1* PCR products used 50% MDE gels (Lonza, Basel, Switzerland) containing 2% glycerol at 15 °C for 16 h at 300 V or 5% glycerol at 15 °C for 20 h at 300 V, followed by silver staining. To sequence the bands separated on the SSCP gels, the polyacrylamide gel was dried after silver staining by sandwiching the gel between Kent paper and a cellophane sheet on an acrylic backplate at 55 °C for 4 h. To extract the DNA, a piece of the DNA band was peeled from the dried gel by using a cutter knife and was incubated in 50 μl of TE buffer (10 mM Tris-HCl and 1 mM EDTA, pH 8.0) at 4 °C overnight. The supernatant solution was used as a template for further PCR amplification with the same primer set employed for the original PCR amplification. Sequence information obtained from voucher materials is provided in Table 1 and the Appendix 1.

**Table 1.** Haplotypes from *trnL-F* and allele *AK1* of D. × kanayamaense, D. × tsukushiense and related species. Any alleles of nuclear gene *AK1* that were identified by sequencing are in boldface. Otherwise, the alleles of nuclear gene *AK1* were deduced from comparisons of band positions in SSCP gels.

**Voucher**	**Species/hybrid**	***trnL-F***	***AK1***	**Locality**
*H.Kanemitsu3746*	D. × kanayamaense	1	**A1A2CK**	Fukuoka Prefecture, Fukuoka City, Sawara-Ku, Mt. Kanayama
*H.Kanemitsu2883*		1	A1A2CK	Fukuoka Pref., Fukuoka City, Sawara-Ku, Mt. Kanayama
*H.Kanemitsu2884*		1	A1A2CK	
*H.Kanemitsu2906*		1	A1A2CK	
*H.Kanemitsu3755*	D. × tsukushiense	1	**A1A2DI**	Fukuoka Pref., Nakagawa City, Ooaza-Gokayama, Tsukushiyabakei
*H.Kanemitsu3756*		1	A1A2DI	
*H.Kanemitsu3757*		1	A1A2DI	
*H.Kanemitsu3750*	D. × toriianum	7	**CHKL1**	Fukuoka Pref., Fukuoka City, Sawara-Ku, Mt. Kanayama
*H.Kanemitsu3751*		7	CHKL1	
*H.Kanemitsu3752*		7	CHKL1	
*K.Hori3023*	*D. chinense*	1	**A1A2**	
*H.Kanemitsu3760*		1	A1A2	Fukuoka Pref., Nakagawa City, Ooaza-Gokayama, Tsukushiyabakei
*H.Kanemitsu3761*		1	A1A2	
*H.Kanemitsu3773*	*D. deciduum*	4	**CHK**	Fukuoka Pref., Fukuoka City, Sawara-Ku, Mt. Kanayama
*H.Kanemitsu3774*		4	CK	
*H.Kanemitsu3775*		4	CHK	
*H.Kanemitsu3892*		4	CHK	
*H.Kanemitsu3893*		4	CHK	
*H.Kanemitsu3905*		4	CHK	
*H.Kanemitsu3914*		4	**CK**	Fukuoka Pref., Fukuoka City, Sawara-Ku, Mt. Nishiyama
*H.Kanemitsu3951*		4	CK	Saga Pref., Saga City, Fuji-Cho
*H.Kanemitsu3758*	*D. fauriei*	5	**DJ**	Fukuoka Pref., Nakagawa City, Ooaza-Gokayama, Tsukushiyabakei
*H.Kanemitsu3759*		5	DJ	
*H.Kanemitsu3992*		5	DJ	
*H.Kanemitsu3881*		5	**DI**	Fukuoka Pref., Iizuka City, Naijukyo
*H.Kanemitsu3989*	*D. squamigerum*	6	**L2**	Fukuoka Pref., Fukuoka City, Sawara-Ku, Mt. Kanayama
*H.Kanemitsu3990*	*D. squamigerum*	6	L2	
*H.Kanemitsu3991*		7	L2	
*K.Hori2336*		4	**B**	Mie Pref., Minamimuro gun, Kiho-Cho, Takaoka
*H.Kanemitsu3753*		4	B	Fukuoka Pref., Fukuoka City, Sawara-Ku, Mt. Kanayama
*H.Kanemitsu3754*		4	B	
*K.Hori3159*	*D. hayatamae*	2	**BF**	Kagoshima Pref., Kumage gun, Yakushima-Cho, Kusugawa
*K.Hori3160*	*D. griffithii*	3	**EG**	

### Molecular analysis

The data set of plastid *trnL-F* phylogeny reflects what we directly sequenced from all the materials. In the dataset of nuclear *AK1* phylogeny, we used all the alleles which we separately picked up from PCR-SSCP gels. Sequences were aligned using MUSCLE (Edgar 2004) and assessed with Bayesian Inference analysis using MrBayes 3.2.6 (Ronquist et al. 2012) and Maximum Parsimony (MP) analysis with MEGA X software (Kumar et al. 2018). Indels were treated as missing characters in the analysis. The best-fit model (*trnL-F*: GTR+G model; *AK1*: HKY+G model) of sequence evolution for DNA regions was selected using jModelTest 2.1.10 (Darriba et al. 2012). Four Markov Chain Monte Carlo loops were run simultaneously and sampled every 100 of 1 million simulations. Tracer 1.7.1 (Rambaut et al. 2018) was used in examining posterior distributions of all parameters and associated statistics, including estimated sample sizes. The first 2500 sample trees from each run were discarded as burn-in periods. An MP tree was obtained using a sub-tree pruning-regrafting algorithm (Swafford et al. 1996) at search level 1, where initial trees were obtained by random addition of sequences (10 replicates). Confidence levels for monophyletic groups were estimated with 1,000 MP bootstrap pseudo-replicates.

## Results

### Plastid and nuclear DNA phylogenetic trees

We sequenced 709–736 bp fragments of the *trnL-F* intergenic spacer from different specimens. The aligned *trnL-F* matrix was 753 bp, of which 140 bp (18%) were parsimony-informative. We then sequenced 280–520 bp of the *AK1* intron for each specimen, yielding a 574 bp aligned matrix, of which 78 bp (13%) were parsimony-informative. The accessions of DNA sequences were listed in Appendix 2.

**Figure 1.**
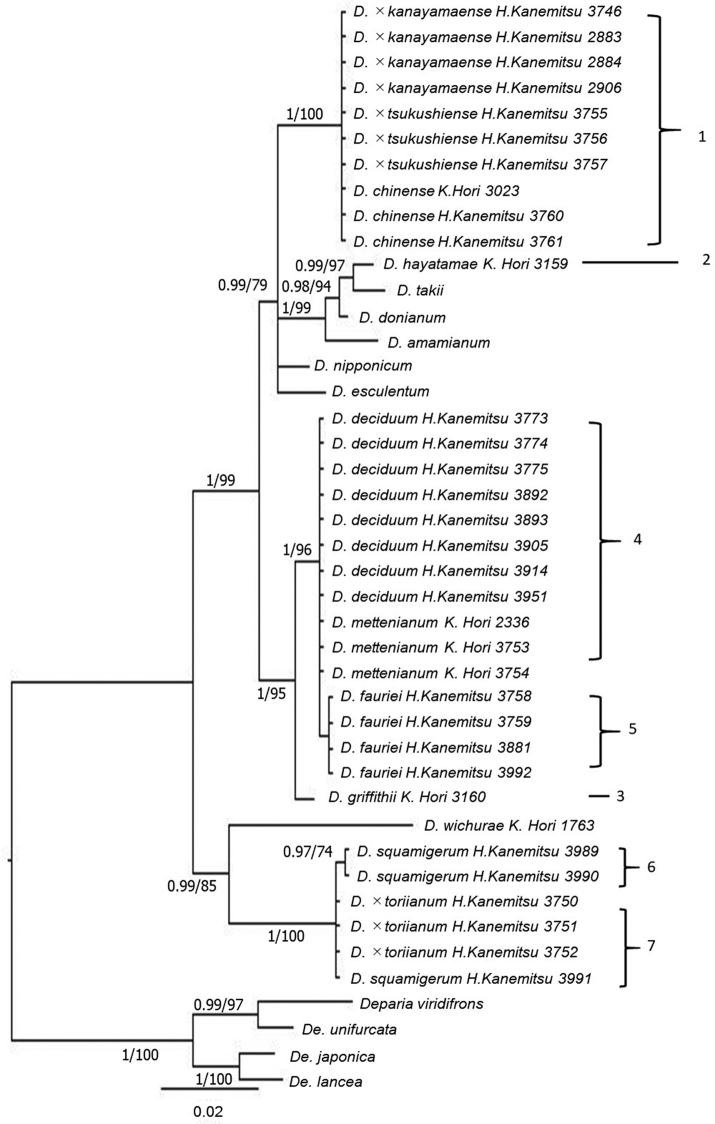
A 50% majority consensus tree resulting from Bayesian Markov Chain Monte Carlo Bayesian (B/MCMC) analysis of plastid *trnL-F* with BI PP (> 0.90) and MP BP (> 70) node support values.

**Figure 2.**
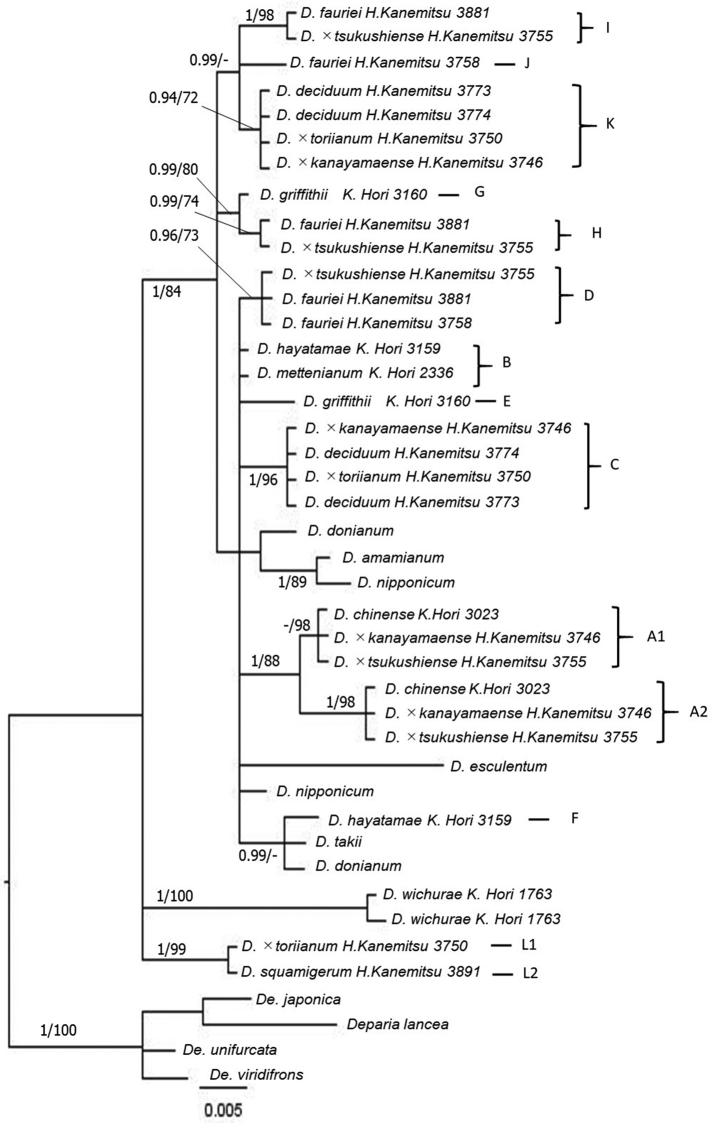
A 50% majority consensus tree resulting from Bayesian Markov Chain Monte Carlo Bayesian (B/MCMC) analysis of nuclear gene *AK1* with BI PP (> 0.90) and MP BP (> 70) node support values.

The 50% majority consensus trees resulting from Bayesian Markov Chain Monte Carlo Bayesian (B/MCMC) analysis of plastid *trnL-F* and nuclear *AK1* gene are shown in Figures 1, 2, respectively.

*Diplazium chinense*, D. × kanayamaense and D. × tsukushiense in *trnL-F* phylogeny displayed haplotype 1. Diplazium × toriianum exhibited the same haplotype 7 as *D. squamigerum*, whereas *D. deciduum* and *D. mettenianum* in the *D. mettenianum* complex showed haplotype 4. Other species in the *D. mettenianum* complex were distinguished in *trnL-F* phylogeny: *D. fauriei*, 5; *D. hayatamae*, 2; and *D. griffithii*, 3. Haplotype 2 of *D. hayatamae* belonged to a different clade than the other species of the complex. *Diplazium hayatamae* has been determined to be closely related to *D. amamianum*, *D. donianum* and *D. takii*.

*Diplazium chinense* had two alleles (A1 and A2) in the same clade in the *AK1* phylogeny. Two clones of *D. deciduum* exhibited two paraphyletic out of three allelic types (CK or CHK). Two clones of *D. fauriei* also exhibited two paraphyletic out of two allelic types (DI or DJ), whereas D. × kanayamaense displayed the same allele of *D. chinense* (A1A2) and one allelic type of *D. deciduum* (CK) completely. Diplazium × tsukushiense displayed the same alleles of *D. chinense* (A1 and A2) and one allelic type of *D. fauriei* (DJ). Diplazium × toriianum had the allele L1 which is closely related to allele L2 of *D. squamigerum* and the three alleles of one allelic type *D. deciduum* (CHK). Furthermore, other species of the *D. mettenianum* complex exhibited different alleles: *D. mettenianum*, B; *D. hayatamae*, BF; and *D. griffithii*, EG.

If the hybrids partly (or incompletely) shared the nuclear DNA allele of parents (in such a case, the hybrid had only nuclear allele A1A2C, A1A2K, A1A2H, A1A2I etc.), we need to assume the relationships between unknown species and present hybrids. In *D. chinense*, there was only one allelic type. There were different allelic combinations in *Diplazium fauriei* (DJ or DI) and *D. deciduum* (CK or CHK). However, the allele of hybrids (D. × kanayamaense: A1A2CK; D. × tsukushiense: A1A2DI) matched either allelic combination of two individuals of *D. deciduum* (*H. Kanemitsu 3914*, *3951*, CK) and one individual of *D. fauriei* (*H. Kanemitsu 3881*, DI) completely. This means we can simply interpret the origin of hybrids to be *D. chinense*, *D. deciduum* and *D. fauriei*. In addition, there are no morphological differences between different allelic types of *D. deciduum* and *D. fauriei*.

Haplotypes of *trnL-F* suggest that one maternal parent of the two new hybrids was *D. chinense*. Alleles in species in the *D. mettenianum* complex were variable and no species of the complex composed a monophyletic group. This might mean members of the *D. mettenianum* complex are allopolyploid or they have incomplete lineage sorting. However, allelic constituents of hybrids suggest that D. × kanayamaense originated as a hybrid of *D. chinense* and *D. deciduum* and that D. × tsukushiense originated as a hybrid of *D. chinense* and *D. fauriei*. These combinations are concordant with intermediate morphological characteristics between likely parents.

### Taxonomic treatment

#### Diplazium × kanayamaense

Taxon classificationPlantaePolypodialesAthyriaceae

K. Hori & H. Kanemitsu hyb. nov.

D3F879F9-7BD7-5853-89A8-CA41A31B007C

Figure 3

##### Diagnosis.

Diplazium × kanayamaense has been determined to be similar to D. × toriianum in having 1-pinnate pinnatifid pinnae curved to an apex. However, lobes of D. × toriianum are obtuse at the apex and scales are more entire on the margin. In contrast, lobes of D. × kanayamaense are acute at the apex and scales show small projections on their margins.

**Figure 3.**
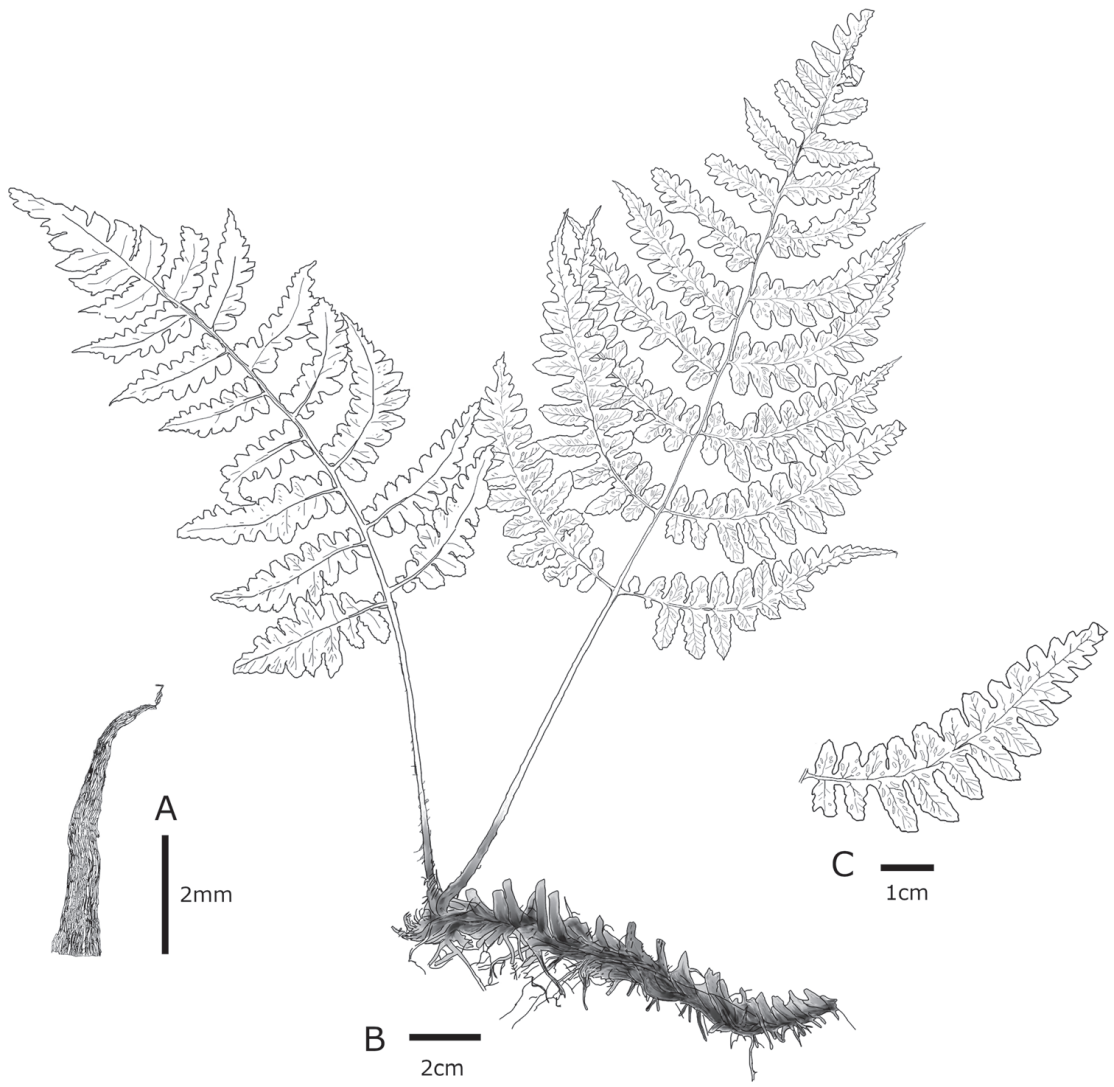
Diplazium × kanayamaense K.Hori & H.Kanemitsu **A** lower stipe scale **B** habit **C** detail of abaxial pinnule **A–C** from the holotype (KAG151589 (illustration by K. Hori).

##### Type.

Japan. Kyushu: Fukuoka Prefecture, Fukuoka City, Sawara-ku, Mt. Kanayama, 33°28'35.89"N, 130°19'23.57"E, alt. 700 m, semi-evergreen forest near streams containing *Carpinus laxiflora* (Siebold et Zucc.) Blume, *Neolitsea sericea* (Blume) Koidz., *Quercus acuta* Thunb and *Stewartia pseudocamellia* Maxim., on soil, 4 Jul 2020, *H. Kanemitsu 3746* (holotype: KAG 151589).

##### Description.

*Terrestrial summergreen fern*. *Rhizomes*: creeping, non-branched, black, 10–15 × 0.5–0.8 cm in diam., closely set with roots and persistent, densely clothed with old stipe bases, glabrous; *fronds*: 2–5 per rhizome; *stipes*: purplish-green, 8–11 × 0.2–0.3 cm in diam., glabrous in middle to upper sections, sparsely clothed with dark brown scales (2.0–4.0 × 0.5–1.0 mm, with small projection on margin) of basal sections, lanceolate; *blades*: fresh green on adaxial surface, 1-pinnate pinnatifid, 1-pinnate at the apex, 15–26.5 × 8–23 cm, ovate; *rachises*: purplish-green, glabrous; *pinnae*: 9–10 pairs, ascending, lanceolate, alternate or opposite, petiolated (2–4 mm long), serrate to lobed, curved from base to apex, acute at the apex, sessile near the apex of blades, widely spaced, lowest pair slightly reduced or the same as second, second lowest pair usually largest, 15–17 cm × 1.5–3 cm; *pinnules*: alternate, 9–10 pairs on the basal sections of the blade, reduced distally, ovate to lanceolate, entirely to shallowly serrated, acute at apex in basal part of blade, obtuse at the apex in the middle section of blades, vein-free, single or double, close to or reaching to the margin, 5–7 pairs in the middle lobe; *the most basiscopic pinnules on the lowest pinnae*: occasionally absent, clearly short, independent from the costa, 2–10 mm × 1.5–4.0 mm; *sori*: long linear- or J-shaped, 1.0–3.0 mm long, on the middle of veinlets, 4–10 pairs per ultimate segment, persistent; *indusia*: cloudy white or brown, same shape as sori, entire, persistent; *spores*: absent or irregular-shaped, abortive.

##### Etymology.

The name derives from Mt. Kanayama, Sawara-ku, Fukuoka City, Fukuoka Prefecture, west Japan, where Diplazium × kanayamaense was initially found.

##### Specimens examined.

**Japan. Kyushu**: Fukuoka Prefecture, Fukuoka City, Sawara-ku, Mt. Kanayama, 33°28'35.89"N, 130°19'23.57"E, alt. 700 m, semi-evergreen forest near streams containing *Carpinus laxiflora* (Siebold et Zucc.) Blume, *Neolitsea sericea* (Blume) Koidz., *Quercus acuta* Thunb and *Stewartia pseudocamellia* Maxim., on soil, 15 Jul 2018, *H. Kanemitsu 2883* (TNS1307641), *H. Kanemitsu 2884* (TNS1307641), *H. Kanemitsu 2906* (TNS1307645).

##### Distribution and ecology.

Diplazium × kanayamaense has been identified to be from Kyushu, western Japan (Figures 3, 5). The species is observed on soil under semi-evergreen forest near streams. This hybrid is endemic to Japan and exists in a population of approximately 124 individuals with juveniles, although parents, *D. chinense* and *D. deciduum*, were both absent near its side.

##### Conservation status.

*IUCN Red List Category.* Based on estimates from GeoCAT, the EOO of D. × kanayamaense was 0.002 km^2^. AOO of D. × kanayamaense was 4.0 km^2^. There were only approximately 124 individuals in the type locality and the population size is not decreasing. According to IUCN (2012) criteria, this hybrid is endangered (EN). A formal evaluation of endangerment can be summarised by the following IUCN hierarchical alphanumeric coding system of criteria: EN D.

#### Diplazium × tsukushiense

Taxon classificationPlantaePolypodialesAthyriaceae

K.Hori & H.Kanemitsu hyb. nov.

A44F3F71-1AD2-58F6-A169-E9A2B0F48B08

Figure 4

##### Diagnosis.

D. × tsukushiense is likened to *D. fauriei* with fronds 1-pinnate at the apex. However, lower pinnae of *D. fauriei* are not lobed, finely serrated on the margin. In contrast, lower pinnae of D. × tsukushiense are lobed deeply and 1-pinnate pinnatifid.

**Figure 4.**
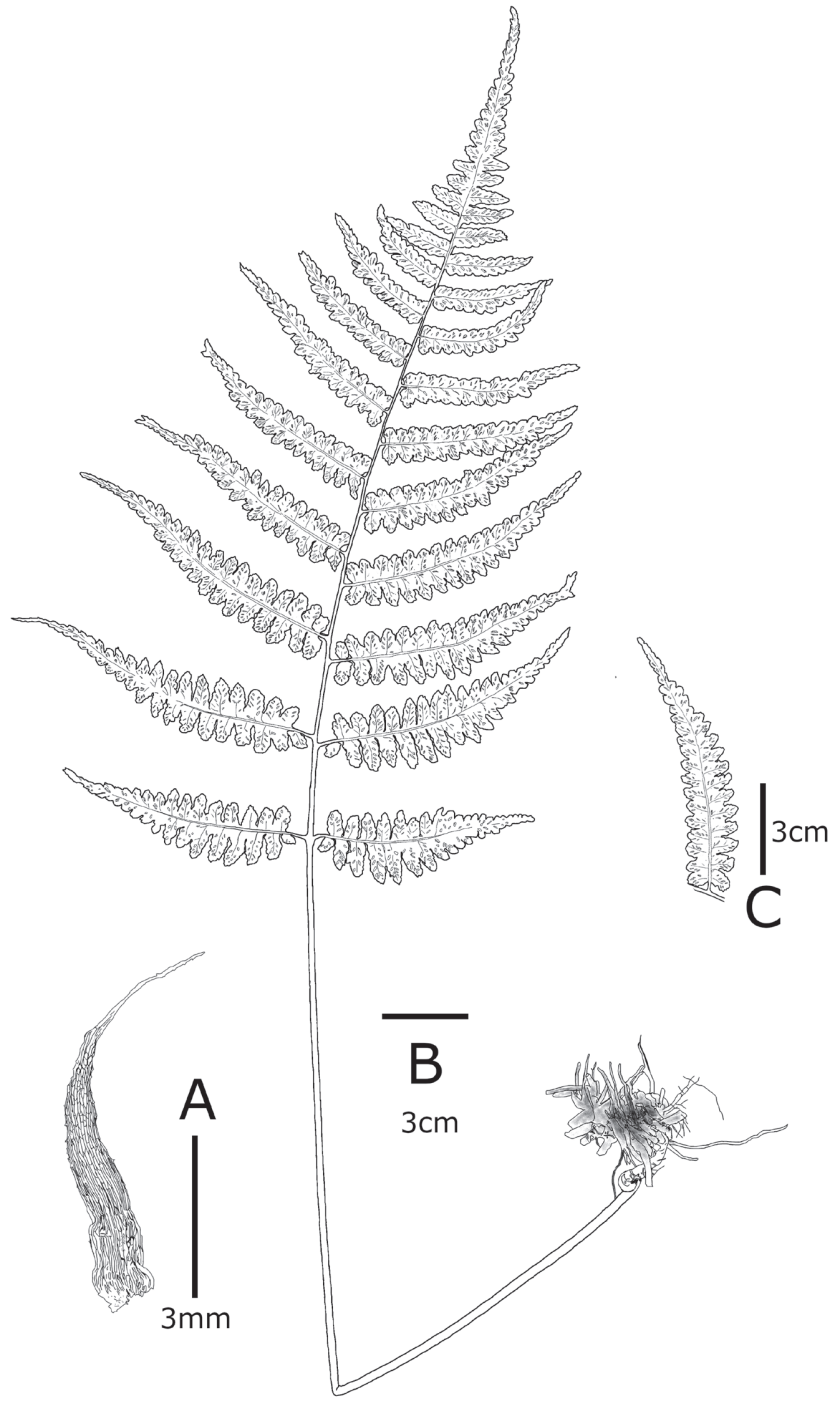
Diplazium × tsukushiense K.Hori & H.Kanemitsu **A** lower stipe scale **B** habit **C** detail of abaxial pinnule **A–C** from the holotype (KAG151590) (illustration by K. Hori).

##### Type.

Japan. Kyushu: Fukuoka Prefecture, Nakagawa City, Ooaza-Gokayama, Tsukushiyabakei, 33°26'22.87"N, 130°25'36.76"E, alt. 266 m, planted coniferous forest containing *Cryptomeria japonica* (Thunb. ex L.f.) D.Don, on soil, 4 Jul 2020, *H. Kanemitsu 3755* (holotype: KAG 151590),

##### Description.

*Terrestrial semi-evergreen fern*. *Rhizomes*: creeping, occasionally two-branched, black, 7–30 cm × 0.8–1.3 cm in diam., closely set with roots and persistent, densely clothed with old stipe bases, glabrous; *fronds*: 2–5 per rhizome; *stipes*: purplish-green, 20–30 cm × 0.2–0.3 cm in diam., glabrous in the middle to upper sections, sparsely clothed with dark brown scales (3.0–6.0 mm × 1.0–1.5 mm, with small projection on margin) on basal sections, lanceolate; *blades*: dark green on adaxial surface, 1-pinnate at the apex, 1-pinnate pinnatifid at the base and middle, 30.0–43.5 cm × 20.0–26.0 cm, narrowly ovate; *rachises*: purplish-green, glabrous; *pinnae*: 10–15 pairs, ascending, straight, lanceolate, alternate, petiolated (2–11 mm long), serrate to lobed, acute at the apex, sessile near the apex of blades, widely spaced, lowest pair of pinnae slightly reduced, second lowest pair usually largest, 10–16 cm × 2.0–5.0 cm; *pinnules*: alternate, 10–15 pairs on the basal sections of the blade, reduced distally, ovate to lanceolate, entirely to shallowly serrated, acute at apex in basal part of blade, rather acute at apex in middle part of blade, vein-free, single or double, close to or reaching to the margin, 5–7 pairs in the middle lobe; *the most basiscopic pinnules on the lowest pinnae*: occasionally absent, slightly short, rather independent from the costa, 3–7 mm × 3–4 mm; *sori*: long linear- or J-shaped, 1.0–5.0 mm long on the middle of veinlets, 4–10 pairs per ultimate segment, persistent; *indusia*: cloudy white or brown, same shape as sori, entire, persistent; *spores*: absent or irregular-shaped, abortive.

##### Etymology.

The name derives from Tsukushiyabakei, Ooaza-Gokayama, Nakagawa City, Fukuoka Prefecture, west Japan, where Diplazium × tsukushiense was initially found.

##### Specimens examined.

**Japan. Kyushu**: Fukuoka Prefecture, Nakagawa City, Ooaza-Gokayama, Tsukushiyabakei, 33°26'22.87"N, 130°25'36.76"E, alt. 266 m, planted coniferous forest containing *Cryptomeria japonica* (Thunb. ex L.f.) D.Don, on soil, 4 Jul 2020, *H. Kanemitsu 3756*, *H. Kanemitsu 3757*, *loc.cit.*, 16 Jul 2018, *H. Kanemitsu 2930* (TNS1307651), *loc.cit.*, 12 Dec 1976, *S. Tsutsui 13341-3* (TNS341595), *loc.cit.*, 8 Jul 1978, *S. Tsutsui 15807* (TNS424776), *loc.cit.*, 6 Nov 1999, *Y. Inoue Y-37* (TNS1159163), *loc.cit.*, 11 Aug 1986, coll. by *T. Yamanaka* (TNS1135951), *loc.cit.*, 11 Aug 1986, coll. by *T. Yamanaka* (TNS1135952), *loc.cit.*, 11 Aug 1986, coll. by *T. Yamanaka* (TNS1135953), *loc.cit.*, 11 Aug 1986, coll. by *T. Yamanaka* (TNS1135954).

##### Distribution and ecology.

**Figure 5.**
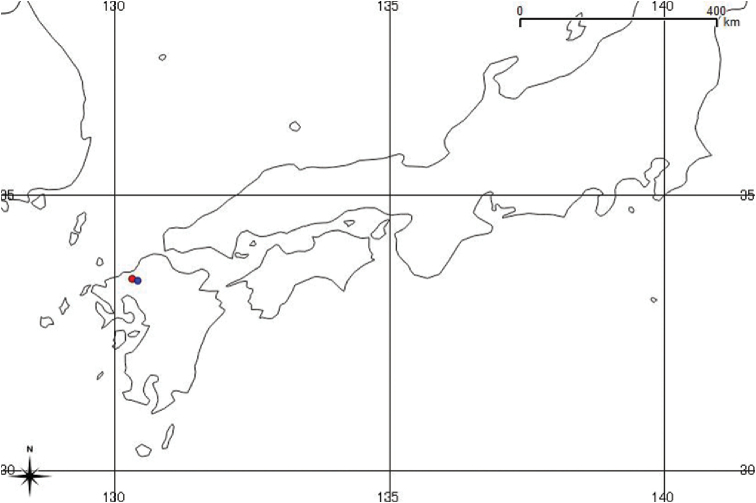
Map showing the known distribution in Japan of D. × kanayamaense = Red circle and D. × tsukushiense = Blue circle.

D. × tsukushiense has been determined to be from Kyushu, western Japan (Figures 4, 5). Observed to grow on soil under coniferous forest containing *Cryptomeria japonica* (Thunb. ex L.f.) D.Don near streams. This hybrid is endemic to Japan. In the type locality, the population is only approximately 10 individuals. Parents *D. chinense* and *D. fauriei* are observed in the same locality.

##### Conservation status.

*IUCN Red List Category.* Based on estimates from GeoCAT, the EOO of D. × tsukushiense is 0.001 km^2^. The known AOO of D. × tsukushiense is 4.0 km^2^. Only 10 individuals are found in the type locality and population size is decreasing because of illegal waste dumping in forests. Therefore, this hybrid should be considered critically endangered (CR), as per the IUCN (2012) criteria. A formal evaluation of endangerment is summarised by the following IUCN hierarchical alphanumeric coding: CR B1ab (i, iv, v)+B2ab (i, iv,v)+C1+C2 a (i, ii) b+D.

## Discussion

The parents of D. × kanayamaense and D. × tsukushiense have been determined to be *D. chinense*, *D. deciduum* and *D. fauriei*. These three species are rather common in western and southern Japan. Therefore, hybridisation amongst these three species is natural to occur more frequently. However, the distribution of D. × kanayamaense and D. × tsukushiense was very narrow in the northern part of Kyushu. We suppose mixed large populations of parents and environmental conditions supported the establishment of hybridisation in the northern part of Kyushu.

We also found that there were differences between the distribution of hybrids and parents. In the type locality of D. × tsukushiense, the allelic composition of *D. fauriei* did not match D. × tsukushiense. We surveyed a wide area around type localities, but eventually, we found the parental individual (allelic type, DI) of *D. fauriei* in a location 30 km away from the type locality of D. × tsukushiense. The difference in the distribution of parents and hybrids suggested hybridisation can decrease or cause the extinction of populations of parents.

**Figure 6.**
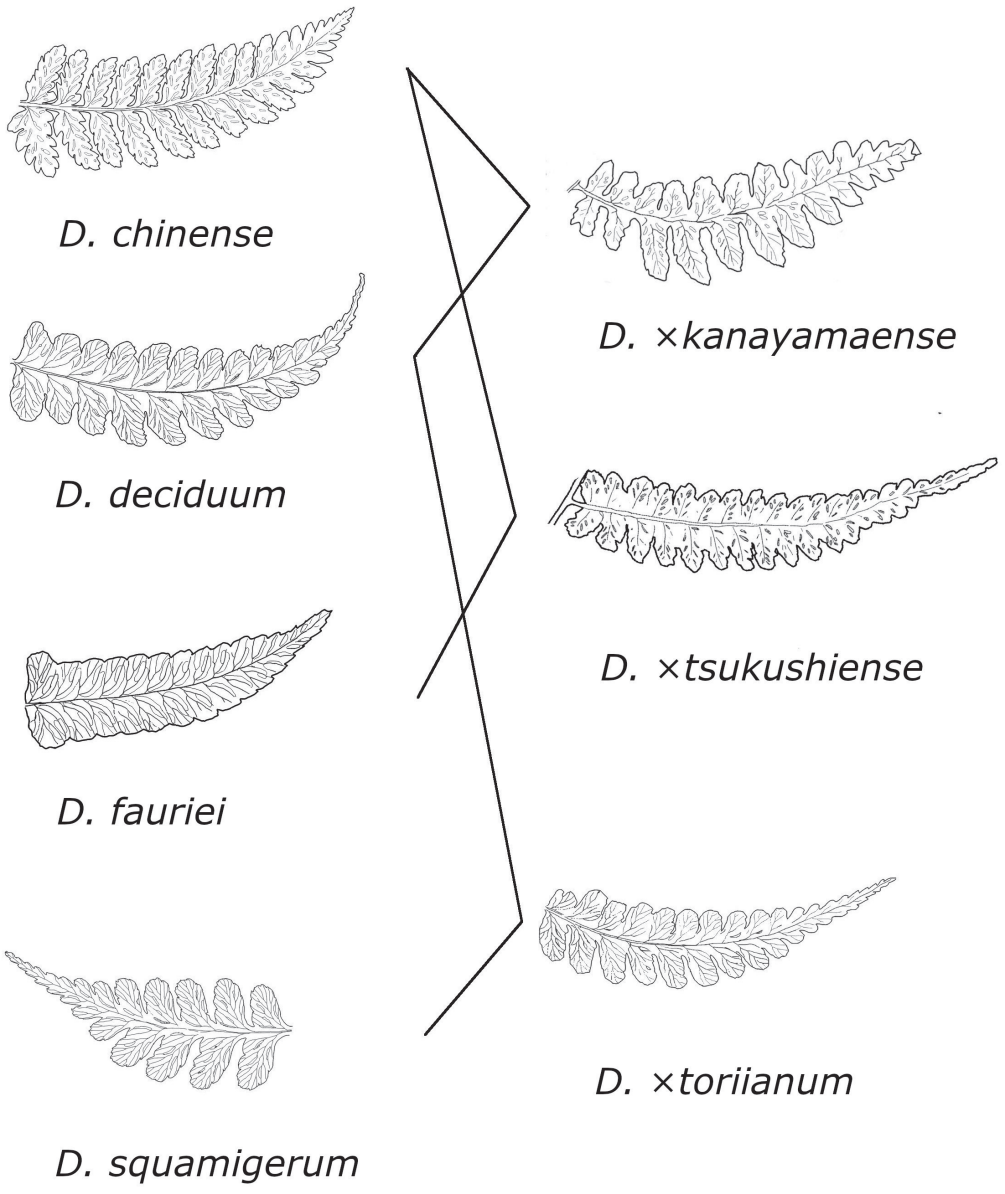
Abaxial surface of pinnae of *D. chinense*, *D. deciduum*, *D. fauriei*, *D. squamigerum*, D. × kanayamaense, D. × tsukushiense and D. × toriianum with relationships of hybrids (illustration by K. Hori).

This study could not estimate the ploidy level of these hybrids because of the difficulty of cultivation. However, for parents of these hybrids, previous cytological studies were well studied by using enough individuals, including type locality and around areas of hybrids (Ohta and Takamiya 1999; Takamiya et al. 2000). Previous cytological studies reported ploidy levels and reproductive modes of parents as follows: *D. chinense*, diploid sexual (Mitui 1968) or tetraploid sexual (Takamiya et al. 2000); *D. deciduum*, hexaploid sexual (Ohta and Takamiya 1999; Takamiya 2006); and *D. fauriei*, tetraploid sexual or hexaploid sexual (Ohta and Takamiya 1999; Takamiya 2006). In addition, Takamiya (2006) reported D. × tsukushiense (*D. chinense* × *D. fauriei*) as a tetraploid sterile. Therefore, hexaploid *D. fariei* had no relationship with D. × tsukushiense. Our materials can be also tetraploid sterile because we collected samples from the same place as Takamiya (2006). We assumed that the ploidy level of D. × kanayamaense can be pentaploid sterile, based on ploidy levels of *D. chinense* (tetraploid) and *D. deciduum* (hexaploid). We do not expect the existence of diploid *D. chinense* because Takamiya et al. (2000) showed enough cytological data of tetraploid *D. chinense*, which were derived from the populations that were sampled across the distribution range of *D. chinense* in Japan. We show the relationships of D. × kanayamaense, D. × tsukushiense and its relatives in Figure 6.

The respective plant size of D. × kanayamaense and D. × tsukushiense shows different characteristics. Diplazium × kanayamaense is smaller than its parents *D. chinense* and *D. deciduum*, but D. × tsukushiense is intermediate between *D. chinense* and *D. fauriei* (Table 2). In D. × kanayamaense and D. × tsukushiense, roots and rhizome both seem to be too weak to survive and difficult to cultivate, especially as most individuals of D. × kanayamaense are juvenile, which are 10 cm tall or less. Therefore, environmental stability is important to maintain individual fern hybrids. The locality of D. × kanayamaense has remained unchanged for years, whereas the locality of D. × tsukushiense seemed to be altered due to illegal dumping activities. Thus, we expect that the discovery of these two new hybrids can assist the conservation efforts for Japanese fern flora.

**Table 2.** Morphological comparison amongst D. × kanayamaense, D. × tsukushiense and related species.

Characteristics	Summergreen/evergreen	Shape of blade	Serration of blade at the base	Apex of pinnules in the basal part of blades	Size of blades (L: long, W: wide)
*D. chinense*	summergreen	deltoid	2-pinnate pinnatifid	acute	40.0–50.0 cm (L) 30.0–40.0 cm(W)
*D. deciduum*	summergreen	ovate	1-pinnate pinnatifid	obtuse	30.2–38.0 cm (L) 20.5–26.0 cm(W)
*D. fauriei*	evergreen	lanceolate	1-pinnate	acute	20.0–30.0 cm (L) 7.0–12.0 cm(W)
*D. squamigerum*	summergreen	ovate	1-pinnate pinnatifid	obtuse	30.0–40.0 cm (L) 25.0–35.0 cm(W)
D. × kanayamaense	summergreen	ovate	1-pinnate pinnatifid	acute	15.0–26.5cm(L) 8.0–23.0 cm(W)
D. × tsukushiense	semi-evergreen	narrowly ovate	1-pinnate pinnatifid	acute	30.0–43.5 cm (L) 20.0–26.0 cm (W)
D. × toriianum	summergreen	broadly ovate or ovate	1-pinnate pinnatifid	obtuse	21.3–22.3 cm (L) 17.5–20.5 cm (W)

## Supplementary Material

XML Treatment for Diplazium × kanayamaense

XML Treatment for Diplazium × tsukushiense

## Appendix 1

Voucher specimens for DNA analysis in this study. **Data are in the order: Species name – locality voucher (Herbarium); haplotype of plastid *trnL-F*; allele of nuclear *AK1*.**

***Diplazium × kanayamaense* K.Hori & H.Kanemitsu**– JAPAN. Fukuoka Pref., Fukuoka City, Sawara-ku, Mt. Kanayama, 4 Jul 2020, *H. Kanemitsu 3746* (KAG); 1; A1A2CK. ibid., 15 Jul 2018, *H. Kanemitsu 2883*, *2884*, *2906* (KAG); 1; A1A2CK.

***D. × tsukushiense* K.Hori & H.Kanemitsu**– JAPAN. Fukuoka Pref., Nakagawa City, Ooaza-Gokayama, Tsukushiyabakei, 4 Jul 2020, *H. Kanemitsu 3755*, *3756*, *3757* (KAG); 1; A1A2DI.

***D. × toriianum* Sa.Kurata**– JAPAN. Fukuoka Pref., Fukuoka City, Sawara-ku, Mt. Kanayama, 4 Jul 2020, *H. Kanemitsu 3750*, *3751*, *3752* (KAG); 7; CHKL1.

***D. chinense* (Baker) C.Chr.**– JAPAN. Kochi Pref., Agawa County, Niyodogawa-cho, Iwayagawa, 16 June 2018, *Hori 3023* (MBK, Hori and Murakami 2019); 1; A1A2. Fukuoka Pref., Nakagawa City, Ooaza-Gokayama, Tsukushiyabakei, 4 Jul 2020, *H. Kanemitsu 3760*, *3761* (KAG); 1; A1A2.

***D. deciduum* N.Ohta & M.Takamiya**– JAPAN. Fukuoka Pref., Fukuoka City, Sawara-ku, Mt. Kanayama, 11 Jul 2020, *H. Kanemitsu 3773* (KAG); 4; CHK. ibid., *H. Kanemitsu 3774* (KAG); 4; CK. ibid., *H. Kanemitsu 3775* (KAG); 4; CHK. ibid., 29 Aug 2020, *H. Kanemitsu 3892*, *3893* (KAG); 4; CHK. ibid., 6 Sep 2020, *H. Kanemitsu 3905* (KAG); 4; CHK. ibid., Mt. Nishiyama, 13 Sep 2020, *H. Kanemitsu 3914* (KAG); 4; CK. ibid., Saga Pref., Saga City, Fuji-cho, 22 Sep 2020, *H. Kanemitsu 3951* (KAG); 4; CK.

***D. fauriei* Christ**– JAPAN. Fukuoka Pref., Nakagawa City, Ooaza-Gokayama, Tsukushiyabakei, 4 Jul 2020, *H. Kanemitsu 3758*, *3759*, *3992* (KAG); 5; DJ. ibid., Iizuka City, Naijukyo, 22 Aug 2020, *H. Kanemitsu 3881* (KAG); 5; DI.

***D. squamigerum* (Mett.) Matsum.**– JAPAN. Fukuoka Pref., Fukuoka City, Sawara-ku, Mt. Kanayama, 4 Jul 2020, *H. Kanemitsu 3989*, *3990* (KAG); 6; L2. ibid., *H. Kanemitsu 3991* (KAG); 7; L2.

***D. mettenianum* (Miq.) C.Chr.**– JAPAN. Mie pref., Minamimuro gun, Kiho-cho, Takaoka, 6 Jul 2016, *Hori 2336* (MAK, Hori and Murakami 2019); 4; B. Fukuoka Pref., Fukuoka City, Sawara-ku, Mt. Kanayama, 4 Jul 2020, *H. Kanemitsu 3753*, *3754* (KAG); 4; B.

***D. hayatamae* N.Ohta & M.Takamiya**– JAPAN. Kagoshima Pref., Kumage gun, Yakushima-cho, Kusugawa, 23 Jan 2019, *Hori 3159* (MBK); 2; BF.

***D. griffithii* T.Moore**– JAPAN. Kagoshima Pref., Kumage gun, Yakushima-cho, Kusugawa, 23 Jan 2019, *Hori 3160* (MBK); 3; EG.

***D. amamianum* Tagawa**– JAPAN. Kagoshima Pref., Amami City, Naze, Honchya-touge, 250 m alt., 7 May 2017, *K. Hatake 615* (MBK, Hori and Murakami 2019).

**D. donianum (Mett.) Tardieu var. donianum**– JAPAN. Kagoshima Pref., Amami City, Sumiyou-machi, Nishinakama, *K. Hori 3228* (MBK).

***D. esculentum* (Retz.) Sw.**– JAPAN. Kagoshima Pref., Isa City, Oguchisogi, *H1109* (MBK, Hori and Murakami 2019).

***D. nipponicum* Tagawa**– JAPAN. Mie Pref., Minamimuro County, Kiho-cho, 70 m alt., 135°59'29.5", 33°45'55.2", 6 July 2016, *K. Hori 2339* (MAK, Hori and Murakami 2019).

***D. takii* Sa.Kurata**– JAPAN. Fukuoka Pref., Kasuya County, Hisayama-machi, 140 m alt., 130°32'2.27", 33°40'44.18", 26 May 2018, *K. Hori 2924* (MBK, Hori and Murakami 2019).

***D. wichurae* (Mett.) Diels** – JAPAN. Kanagawa Pref., Zushi City, Jinnmuji, 60 m alt., 139°36'18.19", 35°18'14.71", 14 Apr 2015, *K. Hori 1763* (MAK, Hori and Murakami 2019).

***Deparia viridifrons* (Makino) M.Kato**– JAPAN. Kochi Pref., Takaoka County, Ochi Town, Mt. Yokogura, 30 May 2018, *K. Hori 2971* (MBK, Hori and Murakami 2019).

***De. unifurcata* (Baker) M.Kato**– JAPAN. Kochi Pref., Agawa County, Niyodogawa-cho, Iwayagawa, 16 June 2018, *K. Hori 3021* (MBK, Hori and Murakami 2019).

***De. japonica* (Thunb.) M.Kato**– JAPAN. Kyoto Pref., Sakyo-ku, Kibune, 300 m alt., 135°45'50.79", 35°7'30.85", July 14 2018, *K. Hori 3031* (MBK, Hori and Murakami 2019).

***De. lancea* (Thunb.) Fraser-Jenk.**– JAPAN. Kochi Pref., Takaoka County, Ochi Town, Mt. Yokogura, 25 June 2020, *K. Hori 3378* (MAK).

## Appendix 2

DNA data accession numbers of the obtained nucleotide sequences used for the construction of molecular phylogenetic trees in this study.

< *trnL-F* >

1, LC468193; 2, LC592258; 3, LC592259; 4, LC592260; 5, LC592261; 6, LC592262; 7, LC592263; *Diplazium donianum*, LC592264; *Deparia lancea*, LC592265.

<*AK1*>

A1, LC468179; A2, LC468182; B, LC468178; C, LC592244; D, LC592245; E, LC592246; F, LC592247; G, LC592248; H, LC592249; I, LC592250; J, LC592251; K, LC592252; L1, LC592253; L2, LC592254; *Diplazium donianum*, LC592255, LC592256; *Deparia lancea*, LC592257.
